# Urological dysfunctions in patients with Parkinson’s disease: clues from clinical and non-invasive urological assessment

**DOI:** 10.1186/s12883-018-1151-z

**Published:** 2018-09-20

**Authors:** Francesca Valentino, Tommaso Vincenzo Bartolotta, Giuseppe Cosentino, Sergio Mastrilli, Valentina Arnao, Paolo Aridon, Salvatore Scurria, Alice Pavone, Carlo Pavone, Marco D’Amelio

**Affiliations:** 1 Dipartimento di Biomedicina Sperimentale e Neuroscienze Cliniche (BioNeC), Università degli studi Palermo, Via Gaetano La Loggia 1, 90129 Palermo, Italy; 2 Dipartimento di Biopatologia e Biotecnologie Mediche (DIBIMED), Università degli studi Palermo, Palermo, Italy; 3 Dipartimento Discipline Chirurgiche, Oncologiche e Stomatologiche (DICHIRONS), Università degli studi Palermo, Palermo, Italy; 4 Università degli studi Palermo, Palermo, Italy

**Keywords:** Parkinson’s disease, Non-motor symptoms, Autonomic dysfunction, Urinary symptoms, AUTonomic SCale for outcomes in Parkinson’s disease, SCOPA-AUT

## Abstract

**Background:**

Autonomic nervous system dysfunction, common in patients with Parkinson’s disease (PD), causes significant morbidity and it is correlated with poor quality of life. To assess frequency of urinary symptoms in patients with PD, without conditions known to interfere with urinary function.

**Methods:**

Non-demented PD patients were consecutively enrolled from the outpatients clinic of our department. Scales investigating motor and non-motor symptoms were carried out. Evaluation of urinary dysfunctions was carried out using the AUTonomic Scale for Outcomes in Parkinson’s disease (SCOPA-AUT) questionnaire. Patients underwent noninvasive urological studies (nUS), including uroflowmetry and ultrasound of the urinary tract.

**Results:**

Forty-eight (20 women, 42%) out of 187 PD patients met the inclusion criteria and were enrolled in the study. Mean SCOPA-AUT score was 14.1 ± 6.9 (urinary symptoms subscore 5.2 ± 3.8). Among those evaluated by the SCOPA-AUT scale, the urinary symptoms were among the most common complaints (93.8%). At nUS mean maximum flow rate (Qmax) was 17.9 ± 9.1 ml/s, and mean postvoid residual (PVR) urine volume was 24.4 ± 44.1 ml. Ultrasound investigation documented prostate hypertrophy in 12 male patients (42.8%). Urinary items of the SCOPA-AUT (SCOPA-U subscore) correlated with measures of disease severity only in female patients.

**Conclusion:**

Urinary symptoms and abnormal findings in nUS are common in PD. Though nigrostriatal degeneration might be responsible for urinary symptoms also in the early-intermediate stage of the disease, when urinary dysfunction occurs other medical conditions need to be excluded.

## Background

Growing evidence suggests that non-motor symptoms (NMS) play a very important, and sometimes a decisive role, in influencing the management of Parkinson’s disease (PD).

Autonomic dysfunction affects up to 70–80% of PD patients, including cardiovascular (orthostatic hypotension, cardiac arrhythmias, lower limb edemas), gastrointestinal (constipation, sialorrhea, heartburn), sudomotor (sweating, seborrhea), thermoregulatory (heat or cold intolerance), and urogenital (urgency, incontinence, erectile dysfunction) symptoms [1]. Urinary symptoms (US) may occur even from the earliest premotor stages of PD and are associated with higher motor disability, more severe dopaminergic denervation, and poorer quality of life [2, 3].

The reported frequency of US in patients with PD varies considerably among studies, mainly due to the use of different and often non-validated questionnaires. Moreover, an important issue that has not always received enough attention is that most PD patients are in the age group in which concomitant pathological conditions might affect urinary function, the most common of which in men is an outflow obstruction due to prostatic enlargement.

Despite these considerations, all authors agree that the urinary symptoms are very common in PD, with recent evidence that prevalence of lower urinary tract symptoms may range from 25 to 50% [4]. In the PRIAMO study [5], US were very common (57.3%), and a follow-up study showed their role as early markers of more severe disease progression [3].

The pathophysiological basis of the urinary dysfunction, that mainly consists in a bladder overactivity, is supposed to rely on a dysfunction of the dopamine basal ganglia circuit, which normally suppresses the micturition reflex [4].

In this study we aimed to assess the occurrence of urinary symptoms in a group of patients suffering from PD who did not suffer from diseases known to influence the urinary function. To this aim, we used a validated questionnaire, i.e. the AUTonomic Scale for Outcomes in Parkinson’s disease (SCOPA-AUT) [6], and we performed a urological examination including noninvasive urological investigation (nUS).

## Methods

Diagnosis of PD was made according to the U.K. PD Brain Bank criteria. PD patients suffering from diseases known to influence the urinary function, i.e. cognitive impairment, major depression and other psychiatric disorders, previous stroke, diabetes mellitus, spondylosis, prostate hypertrophy, urinary tract infections, as well as subjects receiving any medication for urinary problems, were excluded from the study. Clinical assessment of PD patients was performed by SCOPA-AUT questionnaire, Hoehn and Yahr (H&Y) scale, Unified Parkinson’s disease rating scale (UPDRS), Beck Depression Inventory (BDI), Neuropsychiatric Inventory (NPI), Parkinson’s Disease Questionnaire (PDQ-39) and Parkinson’s disease sleep scale (PDSS).

The SCOPA-AUT questionnaire [6] includes 6 items investigating bladder storage and voiding dysfunctions. Storage symptoms include urinary urgency, increased daytime and night-time urinary frequency (nocturia), and urge/stress/mixed incontinence. Increased urinary frequency was defined as passing urine 8 or more times per daytime and twice or more per night. Voiding symptoms consist of hesitancy, slow stream, intermittency, straining, feeling of incomplete emptying and urinary retention. Non-invasive urological studies included uroflowmetry and ultrasound of the urinary tract. The quantitative parameters measured during the voiding phase were maximum flow rate (Qmax) and postvoid residual (PVR) urine volume. Qmax values lower than 10 ml/s and PVR values higher that 50 ml were considered abnormal according to reference values of our laboratory, which were also similar to those used in a previous study conducted in PD patients to assess the urinary function [7]. All PD patients completed a 24-h bladder diary before performing nUS. The 24-h diary included recording of the volume and time of each void, as well as the frequency of incontinence following prompted voiding.

The study was approved by the Ethics Committee of the University of Palermo and all participants gave their informed consent to participate to the study.

### Data analysis

Correlations between clinical/instrumental measures and SCOPA-AUT score and urinary items of the SCOPA-AUT (SCOPA-U subscore) were investigated by means of Pearson’s correlation coefficient. Patients were divided into two groups according to the SCOPA-AUT score (above and below the mean).

In order to investigate the unique role of Parkinson’s disease in determining US, we also divided patients in two groups according to the mean SCOPA-U subscore (below and above the mean value) after we excluded those who showed at nUS organic/structural causes responsible of urinary dysfunction. In both cases, differences between groups were assessed by T test. All analyses were performed with 9.2 SAS version.

## Results

At the end of the enrollment period (from January 2013 to December 2015), 187 PD patients (103 males, 59.9%) were screened. Due to inclusion criteria, thirty-eight PD patients with cognitive impairment, 27 patients receiving medication for urinary problems, and 47 patients suffering from diseases known to influence the urinary function were excluded.

Twenty-seven patients refused to participate to the study, mainly because they declined to undergo instrumental investigations required by the study protocol.

Finally, 48 patients (28 males and 20 females) were included in the study (mean age at interview 62.7 ± 10.6; mean disease duration: 6.2 ± 4.2 years). Most of the patients (84%) were on HY stage 1–2 (33.3% stage 1, 51% stage 2, 8.9% stage 3, 6.7% stage 4 and none stage 5). Demographic and clinical characteristics of the patients enrolled are shown in Table 1. Mean SCOPA-AUT score was 14.1 ± 6.9 while mean SCOPA-U subscore was 5.2 ± 3.8.

**Table 1:** demographic and clinical data (means ± SD) of patients with Parkinson’s disease

	All patients	Females (20)	Males (28)	*p*
Age at onset	56.5 ± 10	58.4 ± 12	55.2 ± 8.4	0.4
Age at interview	62.7 ± 10.6	65.4 ± 12	60.8 ± 9.2	0.3
Disease Duration	6.2 ± 4.2	6.8 ± 4.4	5.7 ± 4.2	0.2
Onset				
Tremor	32/48 (66.7%)	13/20 (65%)	19/28 (68%)	0.8
Rigid-akinetic	16/48 (33.3%)	7/20 (35%)	9/28 (32%)	
UPDRS-I	2.3. ± 2.1	2.4 ± 2.1	2.3 ± 2.2	0.9
UPDRS-II	9.8 ± 7.1	9.5 ± 6.6	10 ± 7.0	0.9
UPDRS-III	17. 1 ± 11.4	16.6 ± 8.7	17.5 ± 13.7	0.8
UPDRS tot	29.3 ± 20.1	29 ± 17.9	29.6 ± 21.9	0.9
LED	518.4 ± 398.5	496. 4 ± 411.8	534.2 ± 402.4	0.7
Levodopa	354.6 ± 329.3	343.4 ± 309.8	352.6 ± 347. 9	0.8
Dopa agonists	169.9 ± 129.8	152. 9 ± 130.3	182.1 ± 131.8	0.4
PDSS	112. 9 ± 22	113.8 ± 25.6	112.3 ± 19.3	0.8
MMSE	28 ± 2	28.2 ± 2.2	28 ± 2.0	0.6
BDI	9 ± 7	9.7 ± 7	8.4 ± 7.1	0.5
NPI	10.7 ± 9.5	11.4 ± 10.3	9.9. ± 8.9	0.6
SCOPA-AUT tot	14.1 ± 6.9	15.1 ± 6.3	13.5 ± 7.4	0.4
SCOPA-U subscores	5.2 ± 3.8	4.8 ± 3.6	5.5 ± 4.4	0.5
Maximum flow rate	17.9 ± 9.1	21.9 ± 12	15.4 ± 6	0.03
Postvoid residual	24.4 ± 44.2	18.9 ± 44.4	28.3 ± 44.4	0.5

*UPDRS* Unified Parkinson’s disease rating scale, *LED* levodopa equivalent dose, *PDSS* Parkinson’s disease sleep scale, *MMSE* mini mental state examination, *BDI* Beck Depression Inventory, *NPI* Neuropsychiatric Inventory, *SCOPA-AUT scale* Scale for Outcomes for Parkinson’s disease AUTonomic, *SCOPA-U* (urinary items) subscore

At least one clinical symptom of dysautonomia was found in all PD patients. The most frequently involved SCOPA-AUT domains referred to the gastrointestinal (95.8%) and the urinary (93.8%) system, followed by cardiovascular (43.7%), thermoregulatory (50%), sexual (31.2%), and pupillomotor (25%) dysfunctions. Only three patients did not complain of urinary problems. Most of the patients with urinary symptoms had a storage disorder (91%), i.e. urgency, nocturia and urinary frequency, while few had a voiding disorder (9%). The instrumental examination showed mean Qmax of 17.9 ± 9.1 SD ml/s and mean PVR of 24.4 ± 44.1 SD ml. Ultrasound documented structural abnormalities of the urinary tract due to prostate hypertrophy in 12 out of 28 (42.8%) male patients, while non-significant alterations were found in the group of female subjects (only in a single subject we found leiomyomas of the uterus).

As shown in Table 2, SCOPA-AUT scores significantly correlated with total (Rho 0.40, *p* = 0.005) and motor section (Rho 0,39, *p* = 0.006) UPDRS scores, levodopa dosage (Rho 0.30, *p* = 0.03), PDSS score (Rho − 0,46, *p* = 0.001), BDI score (Rho 0.31, *p* = 0.03), NPI score (Rho 0.35, *p* = 0.01), and with the following dimensions of the PDQ-39 scale: mobility (Rho 0.34, p 0.002), cognition (Rho 0.37, *p* = 0.01) and communication (Rho 0.30, *p* = 0.004). No correlations with findings of nUS (Qmax and PVR) were found. SCOPA-U subscore significantly correlated only with age at enrollment (Rho 0.41, *p* = 0.003) and with the two following dimensions of the PDQ-39: mobility (Rho 0.29, *p* = 0.04) and cognition (Rho 0.30, *p* = 0.04). No significant correlations with other clinical variables were found. In the hypothesis that this was mainly due to the high frequency of prostate hypertrophy in the male population, we also carried out the correlation analysis considering only female patients. In this group of patients, we found that SCOPA-U subscore significantly correlated along with age at study enrollment (Rho 0.48, *p* = 0.03), also with PDSS score (Rho − 0,55, *p* = 0.01), mobility (Rho 0.54, *p* = 0.01) and cognition (Rho 0.44, *p* = 0.05) of the PDQ-39, and total (Rho 0.46, *p* = 0.04) and motor section (Rho 0,52, *p* = 0.02) UPDRS scores.

**Table 2:** Correlation between clinical/instrumental measures, SCOPA-AUT score and SCOPA-U (urinary items) subscore

	SCOPA-AUT total		Females		Males		SCOPA-U subscore		Females		Males	
	ρ di Pearson	*p*	ρ di Pearson	*p*	ρ di Pearson	*p*	ρ di Pearson	*p*	ρ di Pearson	*p*	ρ di Pearson	*p*
Age at interview	0.22	0.13	0.034	0.90	0.35	0.07	0.41	0.003	0.48	0.03	0.43	0.02
Disease duration	0.20	0.18	0.06	0.80	0.27	0.17	0.08	0.60	−0.009	0.10	0.16	0.4
UPDRS-III	0.39	0.006	0.45	0.05	0.38	0.04	0.27	0.06	0.52	0.02	0.17	0.38
UPDRS tot	0.40	0.005	0.41	0.07	0.40	0.04	0.26	0.07	0.46	0.04	0.16	0.41
LED	0.27	0.06	0.09	0.71	0.40	0.04	0.21	0.15	0.25	0.29	0.18	0.36
Levodopa	0.30	0.03	0.09	0.70	0.43	0.02	0.21	0.14	0.25	0.30	0.19	0.33
Dopa agonists	−0.028	0.85	0.06	0.81	−0.06	0.78	0.10	0.94	0.18	0.45	−0.11	0–57
PDSS	−0.46	0.001	−0.50	0.02	−0.037	0.05	0.12	0.41	−0.55	0.01	−0.08	0.67
BDI	0.31	0.03	0.34	0.14	0.29	0.13	0.14	0.35	0.37	0.1	0.01	0.95
NPI	0.35	0.01	0.37	0.10	0.32	0.09	0.23	0.12	0.30	0.2	0.23	0.24
PDQ-39												
Mobility	0.34	0.02	0.31	0.20	0.34	0.08	0.29	0.04	0.54	0.01	0.20	0.31
Social supp	0.15	0.30	0.12	0.61	0.19	0.33	−0.17	0.24	0.28	0.24	−0.01	0.96
Stigma	0.22	0.13	− 0.029	0.90	0.37	0.05	0.18	0.21	−0.21	0.63	0.41	0.03
Daily activity	0.28	0.06	0.90	0.70	0.40	0.04	0.26	0.08	0.27	0.26	0.25	0.20
Well-being	0.22	0.1	0.28	0.23	0.16	0.41	0.63	0.67	0.30	0.20	−0.04	0.82
Communication	0.30	0.04	0.26	0.36	0.34	0.08	0.14	0.34	0.27	0.25	0.7	0.73
Cognition	0.37	0.01	0.27	0.25	0.41	0.03	0.30	0.04	0.44	0.05	0.24	0.22
Bodily discomfort	0.26	0.08	0.40	0.09	0.14	0.47	0.04	0.76	0.11	0.64	0.05	0.79
Qmax	−0.23	0.14	−0.47	0.06	−0.22	0.27	−0,26	0.09	−0.51	0.04	−0.08	0.70
PVR	0.036	0.80	0.10	0.66	0.1	0.99	0.13	0.40	0.18	0.45	0.07	0.73

## Discussion

In our study, though we enrolled patients with PD without documented history of urinary problems, almost all patients complained of urinary symptoms as shown by the SCOPA-U subscore. Noteworthy, the ultrasound investigation revealed a prostatic hypertrophy as a possible cause of urinary symptoms in a considerable number of male patients.

It is now well established that urinary symptoms can be a troublesome aspect of the non-motor spectrum affecting PD patients. Notwithstanding, there is not yet consensus on the nature, severity and temporal occurrence of urinary symptoms among PD patients [3]. This is also due to the difficulty to make a direct comparison between findings of various studies using different and often non-validated questionnaires and scales.

In this study we used the SCOPA-AUT scale, which is an instrument specifically designed for detection of dysautonomic symptoms in PD including 6 items exploring the urinary function [6]. Though questionnaires and scales evaluating non-motor symptoms [8] such as US can be easily applied in PD patients to address important disease-related issues, these tools are unsuitable to detect bladder dysfunctions with sufficient sensitivity and specificity [6]. Thus, all our patients underwent an in-depth urological clinical and instrumental examination to exclude coexistence of other pathological conditions that could be responsible for urological dysfunctions. As a matter of fact, in our study population the ultrasound investigation revealed a prostate hypertrophy in almost half of males. This benign and treatable pathological condition is often underdiagnosed and undertreated in middle and advanced age male population [9]. Therefore, also on the bases of our results, all male patients with PD and urinary symptoms should underwent a careful urological assessment to exclude conditions responsible for bladder outlet obstruction, and avoid the risk to attribute the urinary symptoms exclusively to the PD.

In our study, almost all patients had an autonomic system involvement, though most of them presented with mild to moderate disease severity (33.3% stage 1, 51% stage 2). This is in line with previous literature showing that autonomic dysfunctions may develop also in early stage of disease and, sometimes, can also precede the diagnosis of PD [10]. We also found that the SCOPA-AUT scores significantly correlated with different measures of motor and non-motor impairment according to the evidence that patients with autonomic symptoms may have higher motor and non-motor burden [11]. It is to note, however, that no significant correlations between SCOPA-U subscores and clinical and instrumental measures were found when considering the whole study population, with the exception of PDSS and PDQ-39 mobility and cognition.

This was likely due to the presence of prostate hypertrophy in a significant number of male patients, that could have acted as a confounding factor. Indeed, correlations between SCOPA-U subscore and total and motor UPDRS scores were found when considering exclusively female patients, in accord to the evidence that prevalence and severity of US in PD patients increases with disease severity [4]. Moreover, the finding that the SCOPA-U subscores significantly correlated with age at enrollment, agrees with other reports showing that PD patients with US are significantly older than patients without US [11, 12].

Findings regarding quality of sleep showed that PD patients with urinary dysfunction had lower scores at PDSS scale, and 75% (27/36) of them complained of nocturia. This supports the concept that nocturnal US are possible determinants of worst quality of sleep [13] and is in line with evidence that patients with US have overall higher non-motor burden [11]. Nonetheless, the coexistence of urinary dysfunction with sleep disorders might also be consistent with the brainstem involvement that may occur early in the course of the disease as demonstrated by neuropathological studies [14].

Main limitations of the study are the relatively low numbers of subjects enrolled and the absence of a control group. Indeed, though several studies have yet reported that PD patients have significantly more US than healthy age-matched control subjects [15, 16] the exact role of age-related changes in the urinary function in PD patients deserves to be investigated in future studies. In many clinical situations, it is difficult to determine to what extent PD is contributing to urinary dysfunction in any given individual. Effective treatment of urologic symptoms in these patients it is often difficult.

## Conclusions

In conclusion, our findings suggest that urinary symptoms are common in PD patients, occurring also in early stages of the disease. It is essential however not to forget that abnormal findings in nUS might alternatively explain symptoms that often are erroneously attributed to Parkinson’s disease. Though autonomic dysfunction might be related to nigrostriatal degeneration, urinary dysfunction in patients with PD could be attributed to other medical conditions.

## Abbreviations

BDI: Beck Depression Inventory
H&Y: Hoehn and Yahr
NPI: Neuropsychiatric Inventory
nUS: Non invasive urological studies
PD: Parkinson’s disease
PDQ-39: Parkinson’s Disease Questionnaire
PDSS: Parkinson’s disease sleep scale
PVR: Postvoid residual
Qmax: Maximum flow rate
SCOPA-AUT: AUTonomic SCale for Outcomes in Parkinson’s disease
SCOPA-U: Urinary items of the AUTonomic SCale for Outcomes in Parkinson’s disease
UPDRS: Unified Parkinson’s disease rating scale
